# Workplace Injuries in Thoroughbred Racing: An Analysis of Insurance Payments and Injuries amongst Jockeys in Australia from 2002 to 2010

**DOI:** 10.3390/ani5030390

**Published:** 2015-09-08

**Authors:** Beverley A. Curry, Peta L. Hitchens, Petr Otahal, Lei Si, Andrew J. Palmer

**Affiliations:** Menzies Institute for Medical Research, University of Tasmania, Hobart TAS 7000, Australia; E-Mails: bevcurry@netspace.net.au (B.A.C.); peta.hitchens@slu.se (P.L.H.); petr.otahal@utas.edu.au (P.O.); Lei.Si@utas.edu.au (L.S.)

**Keywords:** injury, jockey, horse, economic, costs, insurance

## Abstract

*Background*: There is no comprehensive study of the costs of horse-related workplace injuries to Australian Thoroughbred racing jockeys. *Objectives*: To analyse the characteristics of insurance payments and horse-related workplace injuries to Australian jockeys during Thoroughbred racing or training. *Methods*: Insurance payments to Australian jockeys and apprentice jockeys as a result of claims for injury were reviewed. The cause and nature of injuries, and the breakdown of payments associated with claims were described. *Results*: The incidence of claims was 2.1/1000 race rides, with an average cost of AUD 9 million/year. Race-day incidents were associated with 39% of claims, but 52% of the total cost. The mean cost of race-day incidents (AUD 33,756) was higher than non-race day incidents (AUD 20,338). Weekly benefits and medical expenses made up the majority of costs of claims. Fractures were the most common injury (29.5%), but head injuries resulting from a fall from a horse had the highest mean cost/claim (AUD 127,127). *Conclusions*: Costs of workplace injuries to the Australian Thoroughbred racing industry have been greatly underestimated because the focus has historically been on incidents that occur on race-days. These findings add to the evidence base for developing strategies to reduce injuries and their associated costs.

## 1. Introduction

Thoroughbred racing is a popular sport and major industry, and makes a substantial contribution to the Australian economy. In 2005–2006 the Thoroughbred racing industry provided over 64,000 full-time-equivalent jobs, generated more than 5 billion Australian dollars (AUD) and contributed more than AUD 1 billion in government taxes to the Australian economy [1]. In the 2009–2010 race season, Australia had 374 race clubs that conducted 2694 race meetings and 19,376 races, with 194,736 starters vying for over AUD 427 million in prize money [2].

In Australia, approximately 1000 jockeys are licensed to ride in races annually [2], and for them, it is a dangerous occupation. Jockeys in Australia experience an average of one fall every 240 rides in flat racing, with a third of such falls resulting in injury [3]. A fall can be catastrophic, resulting in the end of the jockeys’ career or even death [3,4,5] An Australian Jockeys’ Association survey conducted in 2010, reported that, in the 12 months prior to the survey, at least 50% of jockeys who completed the survey had sustained an injury and 40% had experienced a fall that prevented them from riding [6]. As 41% also reported having no private health insurance and 22% no superannuation, many are dependent on coverage from workers’ compensation [6].

As employees of their relevant Principal Racing Authority (PRA), jockeys unable to earn a living because of work-related injury are provided with near-full income replacement for a defined period. Although conditions and entitlements vary between jurisdictions, a lump sum or periodical payment may be provided for permanent impairment, and where injuries result in death, funeral costs and weekly payments for dependents are also provided [7]. The Personal Accident Insurance (PAI) cover for all jockeys and apprentices, introduced in 2009 [8], funded by a 1% levy on the winnings of all Thoroughbred races [9], is now an important safety net for jockeys with low earnings.

Despite the contribution that horse racing makes to the Australian economy, and not withstanding a study of workers’ compensation costs from Victoria [10] and studies in Britain [4,5], there has been no national study of the economic impact of injuries to Australian jockeys. This study of Australian workers compensation authority (*WorkCover*) data, on claims for horse-related injuries to licensed Thoroughbred racing jockeys, was undertaken to provide national data on this topic.

## 2. Methods

### 2.1. Sources of Data

The Australian Thoroughbred racing industry comprises eight PRAs representing each state or territory of Australia: Racing Victoria Limited; Racing New South Wales; Thoroughbred Racing South Australia Limited; Racing Queensland Limited; Racing and Wagering Western Australia; Tasracing; Thoroughbred Racing Northern Territory and Canberra Racing Club.

With permission from the relevant PRA, a *proforma* spreadsheet, requesting information on all workplace insurance claims by licensed Thoroughbred racing jockeys for the period 1 August 2002 to 31 July 2010, was sent to the *WorkCover* authority in each jurisdiction and the national PAI scheme. Information requested included: the age, sex and experience of the jockey (apprentice/jockey/jumps); the date and a description of the incident; the injury sustained, any absence from work and the (direct) costs associated with the claim.

Introduction of workers’ compensation for jockeys was piecemeal across the states and territories of Australia. In Western Australia (WA) and the Northern Territory (NT), workers compensation cover for jockeys was introduced in 2003 and 2004 respectively, while jockeys in Tasmania and South Australia (SA) were not covered until 2007, therefore data for the whole study period were not available for all jurisdictions. In addition, Queensland data for the period 1 July 2004 to 30 June 2005 were not available.

Ethics approval was granted by the Social Sciences Human Research Ethics Committee, University of Tasmania (Reference H0011786).

### 2.2. Analysis

Descriptive analyses of the costs of claims for workplace injuries (*WorkCover* claims) to licensed jockeys and apprentices were performed. Total costs, mean and standard deviations (SD) are reported. In addition, because a few outliers had a great effect on mean values, median and interquartile ranges (IQR) of distribution are also presented.

As only the cost of claims made to the jockeys’ PAI fund were provided for the 2009–2010 season, more detailed analyses of these data were not possible.

The incidence and costs of *WorkCover* claims per race meeting and ride were calculated using denominator data obtained from the Australian Racing Fact Book 2010 [2]. Claim incidence was calculated using the relevant years’ denominators only where a full year of claim data were available.

Claims were stratified according to whether the corresponding incident occurred on a race-day or elsewhere (grouped as ‘race-day’ or ‘other’) and whether it was a consequence of a fall from a horse (grouped as ‘fall’ or ‘non-fall’). The nature and site of injuries were reviewed and in a subset of claims with details available (WA, Queensland, Tasmania and Victoria); the costs associated with each type of claim were reported.

Prior to analyses, payments were adjusted for inflation to 2011 values using the state and territory specific average weekly earnings for full-time adult persons’ ordinary time earnings at August 2011 [11].

Differences between groups were determined using Wilcoxon rank sum test (for medians) and tests of the equality of proportions and one sample students’ *t*-tests for comparison of means, where appropriate. All analyses were conducted with STATA 12.0 (StataCorp, College Station, TX, USA) with statistical significance at *p* < 0.05.

## 3. Results

### 3.1. Overview of Frequency and Cost of Claims

After exclusion of non-horse related claims (n = 43), claims with no cost attached (n = 193) or where misclassification of jockey status was suspected, data from 2817 Australian jockeys’ workers compensation claims and 115 PAI claims were available for the period 1 August 2002 to 31 July 2010.

The number, total annual cost, and mean and median cost of *WorkCover* claims in each state per year are presented in Table 1. With the exception of WA, the cost of claims fluctuated considerably between racing seasons. Overall, the costs of *WorkCover* claims for horse-related workplace injuries to jockeys cost the Thoroughbred racing industry at least AUD 72.1 million for the eight year study period, an average of AUD 8.6 million per annum based on the last three years where claims data were available for all states and territories. Furthermore, in the first year of the Jockeys’ PAI scheme (2009–2010), 100 claims amounting to AUD 3.3 million were made.

Overall, 39.3% of claims and 51.8% of the total cost of claims were associated with race-day incidents. The mean (AUD 33,756; SD 200,543) and median (AUD 4365; IQR 1104–18,379) costs of race-day incidents were higher than non-race day incidents (AUD 20,338; SD 77,160 and median AUD 3172; IQR 855–12,273).

The incidence of claims according to the number of race meetings and rides in each state are presented in Table 2. Based on the data available for the study period, the overall incidence of claims was around 2.1 per 1000 rides (range 1.43–3.13). The incidence of claims associated with race-day and non-race day incidents was significantly lower than average in Victoria (*p* < 0.001), while SA and WA had a significantly higher than average incidence of total (both *p* < 0.001) and race-day claims (*p* < 0.001 and *p* = 0.020 respectively).

Overall, the mean costs of race-day and non-race day incidents over the years of the study were similar. However, between states there were considerable differences in the costs associated with race-day and other associated claims.

### 3.2. Causes of Claims

Where the status of the jockey was known, 21.9% (22.1% race-day, 21.8% other) of compensation claims from Victoria and 17.4% (17.1% race-day and 17.5% other) from SA were made by jumps jockeys. Overall the mean (AUD 45,831; SD 134,720) and median (AUD 6291; IQR 2137–25,156) costs of the 128 claims by jumps racing jockeys was significantly higher (*p* < 0.001) than the 2659 claims by flat racing jockeys (mean AUD 24,672; SD 140,321; median AUD 3515; IQR 903–13,887). Such claims accounted for AUD 5.7 million (19.6%; 15.2% of race-day associated and 25.1% of other claims) and AUD 111,974 (11.3%; 26.8% of race-day associated and 4.5% of others) of the total cost of claims for Victoria and SA respectively.

**Figure 1:**
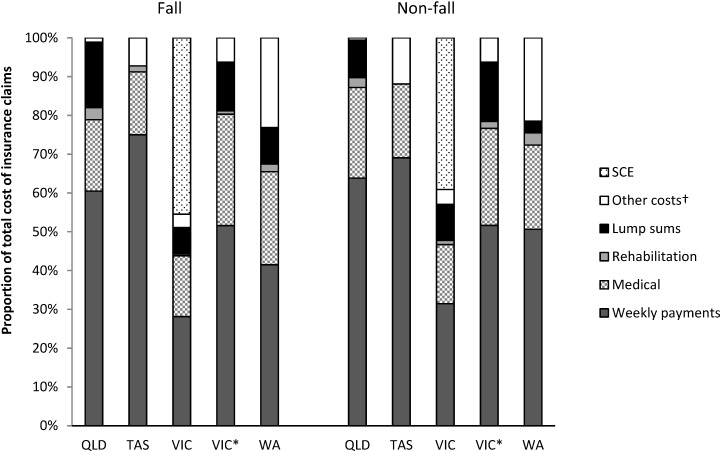
Breakdown of total costs of claims, by jurisdiction, according to whether the claim was a consequence of a fall or non-fall.

The proportion of claims associated with falls from a horse were similar for racing (76.4%) and other riding activities (80.2%) however only 846 (30%) of 1976 claims associated with a jockey fall from a horse only were race-day falls (Figure 1). The mean costs of a race-day fall (AUD 29,250; SD 92,879) was significantly higher (*p* < 0.001) than falls sustained during other riding activities (AUD 16,519; SD 57,082), but there were no significant differences between the median costs (race-day median AUD 5343 and IQR 1060–22,214; other riding median AUD 2811 and IQR 707–10,841). For incidents that did not result in a fall from a horse, there was no statistically significant difference between the mean (AUD 10,399; SD 22,025) or median (AUD 1968; IQR 494–9032) costs of race-day incidents or those occurring during other horse-related activities (mean AUD 10,074; SD 27,290; median AUD 2014; IQR 470–7,773).

QLD = Queensland; TAS = Tasmania; VIC = Victoria; WA = Western Australia;Other costs include legal fees, investigation fees and fees not otherwise specified; Cost breakdown for SA, NT, NSW and ACT unavailable; SCE = Statistical Case Estimate. SCE was included only in Victorian claims; VIC * Victorian data excluding the SCE contribution to the cost.

### 3.3. Injuries Associated with Claims

Where information was available, there were some differences in the causes of injury between race-day and other incidents. Jockey and or horse falls (74.1% *vs.* 67.9%), hitting the barriers or running rail (11.7% *vs.* 4.5%), being kicked or struck (2.7% *vs.* 9.6%), or trampled (1.2% *vs.* 5.4%) by a horse respectively.

**Table 1:** Jockeys’ workplace compensation claims and costs (AUD 1000s), according to Principal Racing Authority and race season.

State		Racing Season								
		2002–2003	2003–2004	2004–2005	2005–2006	2006–2007	2007–2008	2008–2009	2009–2010	Total
NSW	N	158	148	167	99	98	73	101	152	996
	TOTAL COST	3634	3534	3143	1966	1794	2098	2769	2637	21,575
	Mean (SD)	23.0 (96.3)	23.9 (120.1)	18.8 (52.6)	19.9 (57.9)	18.3 (48.9)	28.7 (96.2)	27.7 (105.4)	17.4 (34.1)	21.6 (81.1)
	Median (IQR)	2.6 (0.7,9.7)	2.5 (0.8,9.3)	2.9 (0.7,11.8)	2.4 (0.6,12.9)	2.7 (0.7,14.0)	6.1 (0.4,31.2)	3.0 (0.8,12.3)	4.0 (0.6,17.8)	2.7 (0.7,12.7)
NT	N	N/A	N/A	4	7	7	5	9	10	42
	TOTAL COST			6	82	2168	29	490	126	2901
	Mean(SD)			1.6 (1.6)	11.7 (16.6)	309.8 (607.4)	5.7 (7.0)	54.4 (110.5)	12.6 (12.4)	69.1 (262.0)
	Median(IQR)			1.3 (0.4,2.8)	1.2 (0.4,27.5)	0.8 (0.2,515.4)	1.9 (0.5,9.5)	13.1 (6.9,25.6)	10.0 (2.8,18.1)	5.7 (0.8,18.1)
QLD	N	83	69	N/A	97	112	106	95	86	648
	TOTAL COST	1437	862		2798	1532	2335	1535	986	11,485
	Mean(SD)	17.1 (34.4)	12.5 (19.0)		28.8 (60.6)	13.7 (25.2)	22.0 (39.6)	16.2 (28.7)	11.5 (17.6)	17.7 (35.9)
	Median(IQR)	2.6 (0.9,17.4)	6.6 (1.1,13.0)		4.7 (1.7,24.5)	3.1 (0.8,12.3)	4.8 (0.9,22.2)	3.9 (0.7,13.7)	3.3 (0.6,13.6)	4.0 (0.9,17.2)
SA	N	N/A	N/A	N/A	N/A		28	38	32	98
	TOTAL COST						129	670	231	1030
	Mean(SD)						4.6 (7.6)	17.6 (34.5)	7.2 (9.9)	10.5 (23.1)
	Median (IQR)						1.5 (0.2,5.9)	2.3 (0.8,19.9)	1.6 (0.9,10.5)	1.9 (0.5,9.8)
TAS	N	N/A	N/A	N/A	N/A	N/A	7	9	11	27
	TOTAL COST						71	183	47	301
	Mean (SD)						10.1 (7.2)	20.3 (29.6)	4.3(4.7)	11.2(18.4)
	Median (IQR)						5.9 (4.3,16.8)	8.4 (0.6,26.1)	3.0 (0.9,4.2)	4.2 (0.9,12.5)
VIC	N	82	61	51	63	64	86	70	43	520
	TOTAL COST	8842	1223	2327	829	6949	3985	4821	941	29,917
	Mean (SD)	107.8 (607.5)	20.0 (45.2)	45.6 (134.2)	13.2 (22.5)	108.5 (378.6)	46.3 (103.8)	68.9 (155.7)	21.9 (21.6)	57.5 (288.7)
	Median (IQR)	5.5 (1.3,23.8)	5.9 (2.3,19.9)	8.2 (4.1,33.6)	4.3 (2.2,15.1)	10.0 (3.2,37.9)	5.1 (2.0,33.6)	9.0 (3.7,32.2)	16.1 (8.7,23.8)	7.4 (2.7,23.8)
WA	N	N/A	41	62	92	79	70	76	66	486
	TOTAL COST		624	762	1152	706	668	552	458	4922
	Mean(SD)		15.2 (35.6)	12.3 (40.8)	12.5 (43.3)	10.1(29.3)	9.4 (17.6)	7.2 (13.5)	6.9 (10.8)	10.1 (29.3)
	Median(IQR)		3.5 (0.5,11.3)	1.9 (0.7,5.8)	1.6 (0.7,5.6)	1.9 (0.6,7.1)	1.8 (0.5,7.3)	1.6 (0.5,10.1)	1.9 (0.8,6.6)	1.9 (0.6,7.1)

NSW = New South Wales; NT = Northern Territory; QLD = Queensland; SA = South Australia; TAS = Tasmania; VIC = Victoria; WA = Western Australia; N = number; SD = standard deviation; IQR = interquartile range; NSW data includes data for the ACT; N/A = data not available for time period. AUD, Australian dollars expressed in 2011 values.

**Table 2:** The incidence of workers’ compensation claims and the mean cost per ride, stratified by Principal Racing Authority.

State	Total Rides (N)	Denominator Rides (N)	All Claims			Race-Day Claims			Other Claims		
			N (%)	Claims/1000 rides	Mean cost/ride (SD)	Claims (%)	Claims/1000 rides	Mean cost/ride (SD)	Claims (%)	Claims/1000 rides	Mean cost/ride (SD)
NSW	438,238	438,238	996 (35.4%)	2.3	49 (12)	304 (30.5%)	0.7	19 (8)	692 (69.5%)	1.6	30 (17)
NT	25,702	16,231	42 (1.5%)	2.6	151 (266)	22 (52.4%)	1.4	144 (269)	20 (47.6%)	1.2	7 (8)
QLD	372,453	324,520	648 (23.0%)	2.0	36 (17)	356 (54.9%)	1.1	23 (11)	292 (45.1%)	0.9	14 (8)
SA	117,874	28,600	98 (3.5%)	3.4	24 (20)	35 (35.7%)	1.2	7 (7)	63 (64.3%)	2.2	17 (13)
TAS	49,514	12,064	27 (1.0%)	2.2	16 (12)	12 (44.4%)	1.0	4 (2)	15 (55.5%)	1.2	12 (13)
VIC	370,145	370,145	520 (18.5%)	1.4	80 (63)	197 (37.5%)	0.5	43 (38)	323 (62.1%)	0.9	37 (26)
WA	176,129	154,959	486 (17.3%)	3.1	32 (12)	180 (37.0%)	1.2	16 (6)	306 (63.0%)	2.0	16 (7)
Total	1,550,055	1,344,757	2817 (100%)	2.1	24 (8)	1106 (39.3%)	0.8	12 (5)	1711 (60.7%)	1.3	12 (4)

NSW = New South Wales, including Australian Capital Territory data; NT = Northern territory; QLD = Queensland; SA = South Australia; TAS = Tasmania; VIC = Victoria; WA = Western Australia; N = number; SD = standard deviation; Total rides = The total number of rides during the study period; Denominator rides = The number of rides during the study period with a full season of claims data.; Claims/1000 rides = Number of claims per 1000 rides.; Mean cost/ride = The mean cost of a claim per ride in AUD, calculated where a full year of claim and ride data were available.; All costs expressed as AUD in 2011 values.

A similar proportion of race-day and other claims were associated with strain injuries (4.4% and 5.8%), being crushed or rolled on by a horse (1.7% and 2.1%), being bitten (0.2% and 0.2%), struck by the horses’ head (3.1% and 4.0%) and being dragged by the foot (0.3 and 0.4%), respectively. Where no fall was indicated, three quarters of claims were attributed to jockeys hitting fences or barriers (29.3%), being kicked or struck by a horse (20.1%), strain injuries (20.3%) or being hit by the horses’ head (15.0%). The latter was the main contributor to a significantly greater proportion of claims for facial injuries being associated with non-falls compared to falls (15.0 *vs.* 6.8%, *p* < 0.001). Lower limb injuries were also more frequently associated with incidents where no fall was reported (35.7% *vs.* 26.4%, *p* < 0.001), while falls were associated with more intracranial injuries (5.5% *vs.* 1.8%, *p* < 0.001), neck or shoulder injuries (4.9% *vs.* 2.5%, *p* = 0.001), and multiple injuries (8.2% *vs.* 2.9%, *p* < 0.001). Otherwise, the distribution of other injuries associated with falls and non-falls was comparable: back, 8.0% and 9.9%; chest and trunk, 7.6% and 7.5%; and upper limbs, 30.2% and 24.7%, respectively. Overall, fractures were the most common injury. When further investigated, the proportion of claims attributable to fractures was significantly higher in Victoria (37.3%) and the NT (35.7%) than elsewhere (NSW, 28.4%; QLD, 26.5%; SA, 22.7%; TAS, 14.8% and WA 16.7%). In addition, compared to flat racing jockeys, a greater proportion of jumps racing jockeys’ claims were for fractures (26.1% *vs.* 44.5%, *p* < 0.005).

With the exception of intracranial injuries, where there was a disproportionate high cost for the number of incidents associated with a fall (*p* < 0.001), the distribution of injuries associated with fall and non-fall claims were similar (Table 3) and the median cost of injury claims were of a similar order of magnitude.

### 3.4. Indirect Costs of Injury

Three claims were associated with a fatal injury. Amongst those with non-fatal injuries, the mean absence from work was 45 days (SD 119) and this was greater for jumps racing than flat racing jockeys regardless of whether the incident resulted in a fall (78 days, SD 172 *vs.* 56 days, SD 139 *p* < 0.001) or not (112 days, SD 252 *vs.* 36 days, SD 81, *p* < 0.001). Vertebral fractures and intracranial injuries secondary to a fall were associated with the greatest number of days off work (Table 3).

### 3.5. Breakdown of Direct Costs

Excepting race-day falls in Victoria and WA, which accounted for 44% and 36% of the claims respectively, the majority of the total costs of compensation were for weekly benefits, regardless of the venue or cause of the claim. When documented, around 10% of the total costs of insurance claims were attributed to lump sum payments and 20% to payments for medical services (medical professionals and hospital expenses). Other miscellaneous costs including legal fees, common law payment, investigation costs varied considerably between states. In Victoria, 51% of the total cost of *WorkCover* claims is a Statistical Case Estimate (SCE). The SCE is attributed to each claim to take account of the long-term implications of compensation. After exclusion of SCE from the analysis, the distribution of costs associated with Victorian claims was comparable to other states.

**Table 3:** The number of non-fatal injuries, and total, mean and median costs (AUD 1000s), associated with workers’ compensation claims according to whether or not the injury was sustained as a result of a fall from a horse or where no fall was indicated.

Type of Injury ^a^	Incidents Associated with a Fall					No Fall Indicated				
	N (% Total)	Total Cost (% Overall Cost)	Mean Cost (SD)	Median Cost (IQR)	Days Lost ^a^ (SD)	N (% Total)	Total Costs (% Overall Cost)	Mean Cost (SD)	Median Cost (IQR)	Days Lost ^e^ (SD)
Fractures **^b^**	574 (29.1)	20,674 (35.2)	36.0 (76.1)	14.1 (6.0, 33.6)	74 (109)	194 (23.1)	5137 (38.5)	26.5 (111.5)	8.4 (2.6, 20.3)	50 (87)
Sprains, Strains	399 (20.2)	5843 (9.9)	14.6 (41.0)	2.2 (0.7, 9.4)	29 (88)	136 (16.2)	1424 (10.7)	10.5 (24.3)	2.0 (0.4, 10.5)	26 (71)
Contusion & crushing	348 (17.6)	4781 (8.1)	13.7 (92.8)	1.3 (0.4, 4.0)	24 (141)	142 (16.9)	1024 (7.7)	7.2 (26.4)	1.0 (0.3, 3.2)	22 (90)
Muscle/tendon injury	277 (14.0)	4253 (7.2)	15.4 (54.9)	2.6 (0.8, 6.9)	37 (89)	165 (19.6)	2817 (21.1)	17.1 (59.5)	1.9 (0.7, 8.8)	50 (123)
Intracranial injury **^c^**	105 (5.3)	13,348 (25.0)	127.1 (611.2)	3.0 (1.0, 8.5)	82 (295)	23 (2.7)	105 (0.8)	4.6 (7.1)	1.2 (0.2, 4.4)	14 (33)
Superficial injury	39 (2.0)	281 (0.5)	7.2 (22.9)	0.9 (0.3, 2.8)	17 (54)	19 (2.3)	97 (0.7)	5.1 (8.0)	0.4 (0.2, 12.2)	23 (47)
Vertebral fracture/spinal injury **^d^**	52 (2.6)	3676 (6.3)	70.7 (117.3)	18.2 (6.8, 86.6)	116 (153)	11 (1.3)	1105 (8.3)	100.5 (192.3)	18.0 (4.1, 178.8)	168 (256)
Open Wound, no amputation	26 (1.3)	154 (0.5)	5.9 (10.6)	3.3 (0.8, 5.5)	21 (57)	43 (5.1)	197 (1.5)	4.6 (12.5)	1.3 (0.5, 4.1)	9 (37)
Dislocation	43 (2.2)	1089 (1.9)	25.3 (36.4)	12.2 (3.0, 31.4)	73 (113)	19 (2.3)	497 (3.7)	26.2 (31.3)	9.8 (8.3, 50.1)	63 (109)
Multiple injuries	31 (1.6)	1042 (1.8)	33.6 (62.4)	14.1 (2.9, 37.8)	74 (118)	10 (1.2)	94 (0.7)	9.4 (7.7)	8.2 (3.0, 12.6)	25 (28)
Other and unspecified injuries	78 (3.9)	2323 (4.0)	29.8 (115.0)	2.1 (0.6, 8.8)	35 (101)	79 (9.4)	835 (6.3)	10.6 (45.0)	2.3 (0.4, 8.2)	9 (21)
Overall	1972 (100)	58,799 (100%)	29.8 (160.4)	4.2 (1.1, 17.1)	49 (128)	841 (100)	13,333 (100%)	15.9 (67.6)	2.7 (0.7, 10.2)	35 (97)

**^a^** Excludes identified fatalities (3 Intra cranial injuries, total cost AUD 1,335,659 average cost per claim AUD 445,219 SD 390,253) and one claim for replacement of a personal aid; **^b^** Does not include vertebral fractures; **^c^** Includes concussion; **^d^** Includes 2 spinal injuries: 1 associated with a fall, 1 with no fall indicated; **^e^** Days lost based on 1884 falls and 765 non falls with data available; All costs expressed as AUD × 1000, in 2011 values.

## 4. Discussion

Between 1 August 2002 and 31 July 2010, the direct cost of workplace injuries to jockeys and apprentice jockeys was at least AUD 9 million per annum. Less than half (41%) of the *WorkCover* claims made by jockeys were the result of an incident at a race meeting.

As individual claims had a large effect on the total cost (particularly in smaller jurisdictions), and because of missing data from some jurisdictions, we were unable to evaluate any trends in the costs of insurance claims over time. Differences observed in the incidence and costs of claims between jurisdictions may be related to the different types of racing in each jurisdiction. Only three states conducted jumps racing during the study period (VIC, SA and TAS), and the overall mean cost of claims by jumps jockeys was higher than for flat racing jockeys. This may reflect the severity of injuries experienced by jumps jockeys, and is apparent in the higher cost per claim in Victoria, where most jumps races are held. These findings are consistent with other studies which have found that, although jumps racing jockeys have a higher incidence of falls, and a lower rate of injury per fall [12,13,14], their injuries tend to be more severe. A study of insurance payments to injured jockeys in Great Britain reported that compensation for jumps jockeys was almost twice that of flat racing jockeys [5].

Previous studies of the safety of Victorian race tracks [15,16] identified a number of risk factors for injury and made recommendations on how the incidence of workplace incidents might be reduced. The lower incidence of claims in Victoria relative to other states may be a reflection of the increased focus on injury prevention in that state.

The mean costs of claims resulting from a race-day fall or other incident were higher than those that did not occur on race-day. However, as there were a greater number of non-race day incidents, the overall costs of claims from race-day and non-race day incidents were comparable. This indicates that any estimation of the costs of jockeys’ compensation claims based solely on race-day claims would underestimate the financial burden to the racing industry of injuries to jockeys. This result is in agreement with two other recent investigations of the Victorian Thoroughbred racing industry where about one third of jockey falls were associated with training activities [10,17]. The higher overall incidence of non-race day related claims compared to other studies [10,17] is a new finding. It is possible that that there may have been some misclassification of employment status in our data (70% of insurance claims made to the Victorian *WorkCover* Authority have previously been identified as being made by track riders and stable assistants) [10]. However, our conclusions were unchanged when the analysis was restricted to jurisdictions where jockey status was well characterised (WA).

As observed by others [3,10,17], regardless of whether the incident occurred on a race-day or involved a fall, the most common sites of injury to jockeys were the lower and upper limbs (>49%). Head injuries were less common but they were associated with a higher mean claim costs and more days off work. For incidents that did not result in a fall, facial injuries were also common. As most epidemiological studies of injuries to jockeys concentrate on falls, facial injuries as a consequence of being hit in the head by the horse have not received much attention to date. However, although the costs associated with them might be low, it may be pertinent to consider incorporating facial protection into helmet design in addition to maximizing protection from the impact of a fall.

Of concern are the results from a questionnaire completed by jockeys that indicated that many experience workplace injuries but do not report or make a *WorkCover* claim because a certain amount of injury is accepted as part of the job [17]. In the same questionnaire jockeys reported that they had at least 5 weeks per year off as a consequence of workplace injury [17]. This is comparable with the average absence observed in these data (6.4 weeks) if we assume that each jockey makes only one claim per year.

One of the main limitations to this study of costs is data quality. Inconsistencies in scheme funding, incident documentation, coding methodology and the breakdown of costs associated with claims, apparent in this study, could result in misclassification of incidents.

In response to previous studies highlighting the scarcity of comprehensive data on jockey accidents in Australia [17], an industry database was developed to facilitate standardised documentation of injuries to workers, and horses, at any horse racing facility and improve ascertainment of injury incidence and to identify potential risk factors. When the web-based Australian Racing Injury Database (ARID) was piloted in Victoria and NSW, 115 ARID incident reports were received for the 2008–2009 season [17]. The proportion of these that were race-day events (73%) was in agreement with the number of jockeys in the AJFD in these states (n = 84) who were declared unfit to ride or were taken to hospital after a fall. Combining information from ARID with insurance claims may provide a clearer and consistent picture of the incidence and costs of horse-related injuries to jockey (and other industry workers) throughout Australia.

## 5. Conclusions

Considerable interest and emphasis on the human, equine and monetary costs associated with incidents occurring during races highlights the need to improve safety measures in the horse-racing industry. However, in this study, less than half of the compensation claims were associated with race-day injury. The ARID reporting system may help determine the true incidence of workplace injury in this industry, but monitoring costs associated with workplace injury may provide an additional means of assessing the effectiveness of interventions aimed at risk reduction [15,17].
